# The American Dental Association

**Published:** 1886-06

**Authors:** 


### THE AMERICAN DENTAL ASSOCIATION.

The time for the meeting of the representative dental society of America approaches. Now that all the differences over the selection of a place of meeting are so happily settled, every dentist who has the good of his profession at heart should set himself seriously to work to see in what way he can best aid in the advancement of his calling. If we are to be ranked as a scientific body, we must do scientific work. The real success of the Niagara meeting is to be measured by the technical value of the papers read, and the general tone of the discussions, and not wholly by the numbers in attendance, or the social entertainments offered. The contemplation of elementary subjects should be left to subordinate and local societies. The men who attend the A. D. A. ought not to bring to the meeting crude and undigested thought, nor present rudimentary subjects for consideration. We pray that the members may be spared the long-winded, circumstantial, personal narratives of practice that are too often inflicted upon the listeners at such times. Let every member come prepared with matters of interest, and not trust to the inspiration of the moment for a theme, nor get up and work the pump-handle when the well is too perceptibly dry.

